# Blue–Green Emitting Phosphor Ba_2_LiAlSi_2_O_8_:Eu^2**+**^ for Phosphor-Converted Light-Emitting Diodes via Single-Particle Diagnosis in a Quasi-Quaternary System

**DOI:** 10.1021/acsami.6c02416

**Published:** 2026-05-14

**Authors:** Akihiro Nakanishi, Shiro Funahashi, Yukinori Koyama, Hisanori Yamane, Kohsei Takahashi, Takayuki Nakanishi, Naoto Hirosaki, Takashi Takeda

**Affiliations:** † Advanced Phosphor Group, 543618 National Institute for Materials Science, Tsukuba, Ibaraki 305-0044, Japan; ‡ Center for Basic Research on Materials, National Institute for Materials Science, Tsukuba, Ibaraki 305-0047, Japan

**Keywords:** Eu^2+^-activated phosphor, single-crystal X-ray diffraction, quasi-quaternary system, new crystal phase, phosphor-converted LEDs

## Abstract

Phosphor-converted light-emitting diodes (PC-LEDs) are widely used in various fields due to their long lifetime and high energy efficiency. In particular, blue–green-emitting phosphors have the potential to fill the cyan gap in white LEDs and to be used in display indicators for autonomous driving. A new blue–green-emitting Ba_2_LiAlSi_2_O_8_:Eu^2+^ phosphor was discovered through exploratory experiments in the BaO–Li_2_O–Al_2_O_3_–SiO_2_ quasi-quaternary system using a single-particle-diagnosis approach. Single-crystal X-ray diffraction analysis revealed that Ba_1.96_Eu_0.04_LiAlSi_2_O_8_ crystallizes in a space group of *Pna*2_1_ (No. 33) with *a* = 8.04521(11) Å, *b* = 19.0484(2) Å, *c* = 5.02228(6) Å, and *Z* = 4. The crystal structure comprises LiO_4_, AlO_4_, and SiO_4_ tetrahedra, which orderly align and form a framework by sharing apical oxygen atoms. Ba atoms are surrounded by eight and seven oxygen atoms in the framework. Density functional theory calculations corroborated the Al and Si arrangement in the Ba_2_LiAlSi_2_O_8_ crystal structure. A single-phase powder of the Ba_2_LiAlSi_2_O_8_:Eu^2+^ phosphor was successfully obtained via a solid-state reaction. This phosphor exhibited a blue–green luminescence peak at 497 nm with a full width at half-maximum of 85 nm under 372 nm excitation. The internal and external quantum efficiencies were 51.0% and 42.7%, respectively. The peak intensity at 150 °C was 67% of that at room temperature. We fabricated pc-LEDs based on 405 nm LED chips combined with Ba_2_LiAlSi_2_O_8_:Eu^2+^ phosphor, and the CIE chromaticity coordinates were in the blue–green region. These results indicate that the new Ba_2_LiAlSi_2_O_8_:Eu^2+^ phosphor is a promising candidate for future LED technologies.

## Graphical abstract

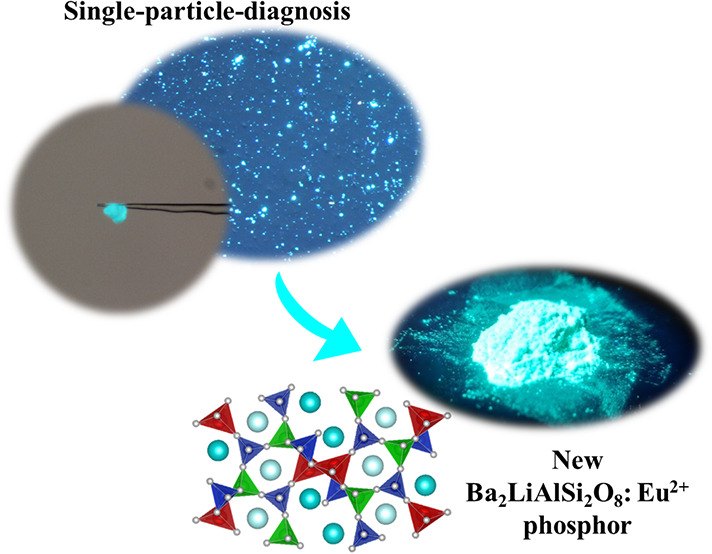

## 1 Introduction

Phosphor-converted light-emitting diodes (pc-LEDs) have applications in various fields due to their advantages of long life and high-energy efficiency.^1–3^ A typical example is the combination of blue-LEDs^4^ and yellow phosphors to create white light.^5^ Color-conversion phosphors are widely studied for various applications. Particularly, yellow-emitting (Y_3_Al_5_O_12_:Ce^3+^ and Ca-α-SiAlON (Ca_*m*/2_Si_12–*m*–*n*_Al_*m*+*n*_O_*n*_N_16–*n*_:Eu^2+^)), blue-emitting (BaMgAl_10_O_17_:Eu^2+^), and red-emitting (CaAlSiN_3_:Eu^2+^ and *M*_2_Si_5_N_8_:Eu^2+^ (M = Ca, Sr)) phosphors are commercially available for fabricating white LEDs comprising blue or ultraviolet LED chips.^5–9^ Narrow-band green phosphor β-SiAlON:Eu^2+^ (Si_6–*z*_Al_*z*_O_*z*_N_8–*z*_:Eu^2+^) is used in liquid crystal display backlights to enlarge color gamuts.^10,11^

The development of blue–green emitting phosphors, which are used for developing high color-rendering white LEDs to fill cyan gaps, has attracted considerable attention in the advancement of LED applications.^12,13^ In addition, blue–green lights with wavelengths of 440–570 nm exhibit deep penetration depth in marine environments, resulting in their usage for underwater wireless optical communication, fish attractions, and aquaculture in marine ranching systems.^14,15^ Recently, turquoise-colored exterior marker lights have been approved by Mercedes-Benz for vehicles equipped with its Drive Pilot SAE (Society of Automotive Engineers) Level 3 automated driving system.^16^ Therefore, blue–green emitting phosphors are expected to play a pivotal role in future LED technologies.

Eu^2+^- or Ce^3+^-activated phosphors are attractive for LED applications because of the parity-allowed transition between 5d- and 4f-orbital.^17^ Their luminescence properties are strongly influenced by the local structure surrounding Eu^2+^ or Ce^3+^,^18–21^ and this local structure varies with the host material. Therefore, the discovery of suitable host materials is crucial for accelerating phosphor development. However, identifying new host materials remains time-consuming and costly because no clear guidelines for their discovery have been established so far. Traditionally, phosphor development has relied on a trial-and-error approach, typically using known host materials listed in databases (e.g., Inorganic Crystal Structure Database^22^) or by exhaustively screening possible elemental combinations to find new host materials. We previously proposed a single-particle-diagnosis approach that is highly effective for discovering new phosphors.^23^ This approach enables the direct determination of the crystal structure and luminescence properties of single-crystal phosphors from multiphase powders containing various secondary phases, without the need for single-phase synthesis. Using this approach, new (oxy)­nitride phosphors, such as Ba_5_Si_11_Al_7_N_25_:Eu^2+^, BaSi_4_Al_3_N_9_:Eu^2+^, Ba_2_LiSi_7_AlN_12_:Eu^2+^, Sr_3_Si_8–*x*_Al_*x*_O_7+*x*_N_8–*x*_:Eu^2+^, Ca_1.62_Eu_0.38_Si_5_O_3_N_6_, and Si_2.5_Al_9.5_O_0.5_N_12.5_:Eu^2+^, were discovered.^23–27^

Oxide materials are relatively easy to synthesize; consequently, numerous oxide-based compounds have been reported. To further expand the exploration of oxide materials, exploratory experiments in multicomponent systems combining the single-particle-diagnosis approach offer a more effective strategy for material discovery. In this study, we explored a new oxide-based Eu^2+^-activated phosphor in the BaO–Li_2_O–Al_2_O_3_–SiO_2_ quasi-quaternary system. In previous studies, Eu^2+^ -activated phosphors have been discovered in the Ba–Li–Al–Si–N system using the single-particle-diagnosis approach.^24^ Eu^2+^ -doped Ba_2_SiO_4_, BaAl_2_Si_2_O_8_, BaSiO_3_, Li_2_BaSiO_4_, and BaAl_2_O_4_ have been reported to exhibit blue to yellow emission.^28–32^ However, in the oxide-based Ba–Li–Al–Si–O system, no new phases have yet been discovered, leaving room for further exploration. A new blue–green emitting Ba_2_LiAlSi_2_O_8_:Eu^2+^ phosphor was discovered using the single-particle-diagnosis approach, and a Ba_2_LiAlSi_2_O_8_:Eu^2+^ phosphor powder was successfully synthesized for pc-LED application.

## 2 Experimental Section

### 2.1 Exploratory Synthesis and Characterization of Particles

Various Ba/Eu/Li/Al/Si/O compositions were explored with reagents of BaCO_3_ (99.95%, Kojundo Chemical, Japan), Li_2_CO_3_ (99.99%, Kojundo Chemical, Japan), Al_2_O_3_ (99.99%, TAIMEI CHEMICALS Co., LTD, Japan), SiO_2_ (99.9%, Kojundo Chemical, Japan), and Eu_2_O_3_ (99.9%, Shin-Etsu Chemical Co., LTD, Japan). The mixed precursor powders were calcined on an alumina boat at 1050 °C for 5 h in a reducing atmosphere (H_2_:N_2_ = 5:95 gas). The resulting powders were excited by a 365 nm LED, and a blue–green emitting phosphor particle was collected under microscope observation. Single-crystal X-ray diffraction (XRD) measurements of the particles were performed using a diffractometer (XtaLab Synergy-Custom, Rigaku, Japan) with Mo–Kα radiation (λ = 0.71073 Å). Data were integrated and corrected for absorption using *CrysAlisPro*. The crystal structures were refined by *SHELX*.^33,34^ The chemical compositions were analyzed using a scanning electron microscope (Hitachi High-Technology, SU1510) with an energy dispersive spectroscope (EDS, Bruker AXS, XFlash SDD) operated at 10 kV. The excitation and emission spectra of the particles were measured using a self-made device and analyzed using a proximity method.^35^

### 2.2 Density Functional Theory Calculations to Examine Cation Ordering in Ba_2_LiAlSi_2_O_8_

Density functional theory (DFT) calculations were performed using the plane-wave basis projector augmented wave method, as implemented in the Vienna *Ab initio* simulation package VASP 6.3.2,^36,37^ to examine the arrangement of Li, Al, and Si atoms in Ba_2_LiAlSi_2_O_8_. The Perdew–Burke–Ernzerhof exchange-correlation functional^38^ was used, with the cutoff energy set to 520 eV. Reciprocal-space integration was conducted using a gamma-centered 2 × 2 × 4 mesh. The total energy converged to 10^–9^ eV/atom. The lattice constants and internal coordinates were optimized until the force converged to 0.01 eV/Å.

### 2.3 Powder Synthesis and Characterization of Ba_2_LiAlSi_2_O_8_:Eu^2+^

Ba_2(1–*x*)_Eu_2*x*_LiAlSi_2_O_8_ (*x* = 0, 0.005, 0.02, 0.04, and 0.06) phosphors were synthesized via a solid-state reaction. The stoichiometric amounts of BaCO_3_, Li_2_CO_3_, Al_2_O_3_, SiO_2_, and Eu_2_O_3_ were thoroughly mixed in an alumina mortar. After mixing, the precursor powder was calcined in the alumina boat at 1050 °C for 5 h in a reducing atmosphere (H_2_:N_2_ = 5:95 gas). The crystal phases of the phosphors were analyzed at room temperature via powder XRD (SmartLab X-ray Diffractometer, Rigaku, Japan) with Cu-*Kα*_1_ radiation at 45 kV, 200 mA, and 2θ in the range of 10°–90°. The XRD data were analyzed using Rigaku PDXL 2 software. The luminescence properties of powder samples were measured with a fluorescence spectrometer (FP-8600 Spectrofluorometer, JASCO, Japan), and the decay curves were measured with a fluorescence spectrometer (FLS1000 Spectrofluorometer, Edinburgh Instruments Ltd., UK) under the excitation of a 375 nm pulse laser diode. The internal quantum efficiency (IQE) and external quantum efficiency (EQE) of the phosphors were measured using a QE-2100 system (Otsuka Electronics, Japan). BaSO_4_ was used for a white reference. The cation content was analyzed via inductively coupled plasma optical emission spectroscopy (5800 ICP-OES, Agilent, USA). The measurement solution was prepared by dissolving the sample powder in solution. Sample powder was fused with sodium carbonate and boric acid in a platinum crucible. After cooling, the melt was dissolved in 10 mL of HCl (1 + 1) and diluted with water. The solution was transferred to a flask, and Yb standard solution was added as an internal standard. The temperature-dependent luminescence properties were measured using the QE-2100 system combined with a temperature control stage (10002L, Linkam Scientific Instruments, UK).

### 2.4 Characterization of pc-LED with Ba_2_LiAlSi_2_O_8_: Eu^2+^ Phosphor

The pc-LEDs were fabricated by coating a mixture of the obtained phosphors and silicone resin and with a 405 nm LED chip. Particularly, the obtained phosphors and silicone resin were mixed at a weight ratio of 1:3. The resulting paste mixture was coated on the LED chip and heated at 150 °C for 30 min to cure the resin. The electroluminescence (EL) spectra of the obtained pc-LEDs were measured using the QE-2100 system.

## 3 Results and Discussion

### 3.1 New Phosphor Discovery

A new oxide phosphor was discovered in the product from a composition of Ba/Eu/Li/Al/Si = 39.2:0.8:10:10:40. Figure 1 shows a microscopic photograph of the powder product under 365 nm excitation light. Several particles showed emission in the blue–green region, and some particles with yellow emission were observed. Ba_2_SiO_4_, BaSiO_3_, and BaAl_2_Si_2_O_8_ were identified in the powder XRD analysis (Figure S1). It has been reported that Ba_2_SiO_4_: Eu^2+^ and BaAl_2_Si_2_O_8_: Eu^2+^ phosphors show blue emission^28,29^ and BaSiO_3_: Eu^2+^ shows yellow emission.^30^ However, some XRD peaks were not consistent with known Ba/Li/Al/Si-containing oxides, suggesting it is a new compound. In the single-crystal XRD analysis of the emission particles, a new phosphor with blue–green emission was discovered. Figure S2 shows the excitation and emission spectra of blue–green emitting single particles. The monitored excitation spectrum has a broad band in the near-ultraviolet (UV) region, and the emission spectrum shows an emission peak at 491 nm under 350 nm irradiation.

**Figure 1:**
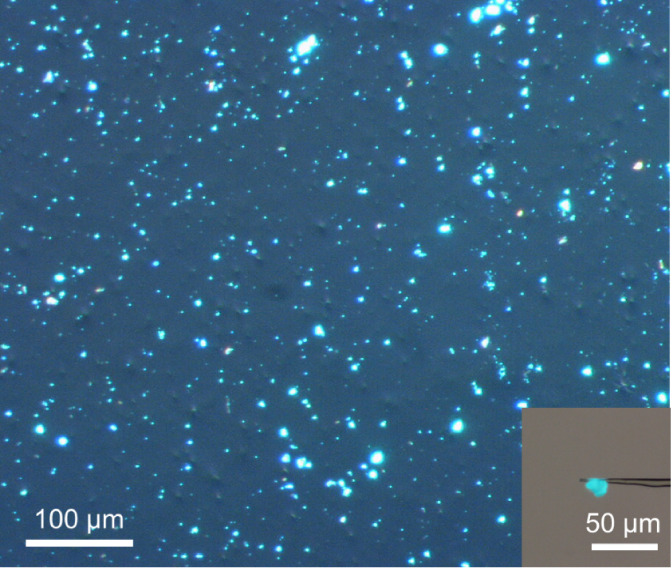
Photograph of a powder product from the composition of Ba/Eu/Li/Al/Si = 39.2:0.8:10:10:40 under 365 nm excitation light. The inset shows the selected blue–green luminescent particle for analysis.

The blue–green emitting particle was determined as a new Ba_2_LiAlSi_2_O_8_ material via the single-crystal XRD analysis. Table 1 lists the crystallographic data and refinement structure parameters. Table 2 lists the fractional coordinates. The Eu occupancy was refined based on the nominal Eu content of 2% relative to Ba, as defined by the starting stoichiometric ratio used in the synthesis. The crystal structure has an orthorhombic system with *a* = 8.04521(11) Å, *b* = 19.0484(2) Å, *c* = 5.02228(6) Å, and a space group of *Pna*2_1_ (No. 33). The reliability factors *R*_1_ and *wR*_2_ were 2.41% and 3.71%, respectively. Table S1 lists the anisotropic displacement parameters. Figure S3 shows the EDS spectrum and analyzed cation ratio of the blue–green emitting single particles. No clear Eu signal was obtained because the amount of Eu is small; thus, it was excluded from the analysis. The atomic ratios of Ba, Al, and Si were 13.6%, 7.2%, and 13.9%, respectively. The cation ratio is in close agreement with the 2:1:2 ratio obtained by single-crystal XRD structural analysis.

**Table 1:** Crystallographic Data and Structure Refinement Parameters of Ba_1.96_Eu_0.04_LiAlSi_2_O_8_ ^a^

Formula	Ba_1.96_Eu_0.04_LiAlSi_2_O_8_
Formula mass (g mol^–1^)	493.36
Crystal system	Orthorhombic
Space group	*Pna*2_1_ (No. 33)
Temperature (K)	293
*a* (Å)	8.04521(11)
*b* (Å)	19.0484(2)
*c* (Å)	5.02228(6)
*V* (Å^3^)	769.659(17)
*Z*	4
Radiation type	Mo *Kα*
μ (mm^–1^)	10.727
θ range for data collection (°)	2.7380 to 38.5940
Index ranges	–13 ≤ *h* ≤ 10, −31 ≤ *k* ≤ 31,–8 ≤ *l* ≤ 8
Crystal size (mm)	0.019 × 0.017 × 0.011
Diffractometer	ROD, Synergy Custom system, HyPix-Arc 150
Absorption correction	Multiscan
*T*_min_, *T*_max_	0.827, 1.000
Reflection collected	55852
Independent reflections	3736
Final *R* indexes [*I* ≥ 2σ (*I*)]	*R*_1_ = 0.0218, *wR*_2_ = 0.0367
Final *R* indexes [all data]	*R*_1_ = 0.0241, *wR*_2_ = 0.0371
*S*	1.204
Δρ_max_, Δρ_min_ (e Å^–3^)	1.07, −1.40

a *R*_1_, *wR*_2_ are reliability factors. *S* is goodness of fit. *R*_1_=*∑*|(|*F_o_* | – |*F_c_* |)|/*∑*|*F_o_* |. $wR_{2}=\{\sum \left[w{\left(F_{o}^{2}-F_{c}^{2}\right)}^{2}\right]/\sum \left[w{\left(F_{o}^{2}\right)}^{2}\right]{\}}^{1/2}$ . $S=\{\sum \left[w\left({\left(F_{o}^{2}-F_{c}^{2}\right)}^{2}\right)\right]/\left(n-p\right){\}}^{1/2}$ . $w=1/\left[\sigma^{2}\left(F_{o}^{2}\right)+{\left(0.009P\right)}^{2}+1.6987P\right]$ . $P=\left[2F_{c}^{2}+\max\left(F_{o}^{2},0\right)\right]/3$ . *F_o_* is the observed structure factor, *F_c_* is the calculated structure factor, *σ* is the standard deviation of $F_{o}^{2}$ , *n* is the number of reflections, and *p* is the number of refined parameters.

**Table 2:** Occupancies, Fractional Atomic Coordinates, and Equivalent Isotropic Atomic Displacement Parameters (*U*_eq_) of Ba_1.96_Eu_0.04_LiAlSi_2_O_8_ ^a^

Atom	Occupancy	*x*	*y*	*z*	*U*_eq_ (Å^2^)
Ba/Eu1	0.979(3)/0.021(3)	0.18003(3)	0.55048(2)	0.37803(5)	0.00919(4)
Ba/Eu2	0.981(3)/0.019(3)	0.47812(2)	0.72172(2)	0.40001(5)	0.00916(4)
Li	1	0.4870(8)	0.5581(4)	0.8649(18)	0.0119(12)
Al	1	0.80188(13)	0.65697(5)	0.8667(3)	0.00543(18)
Si1	1	0.19239(11)	0.68060(4)	0.8825(3)	0.00532(13)
Si2	1	0.71634(12)	0.57203(5)	0.3649(2)	0.00545(16)
O1	1	0.8616(4)	0.51509(14)	0.3463(6)	0.0122(6)
O2	1	0.6950(4)	0.59328(14)	0.6833(5)	0.0075(5)
O3	1	0.7679(4)	0.64613(15)	0.2089(5)	0.0098(5)
O4	1	0.2222(4)	0.76149(14)	0.7714(6)	0.0094(5)
O5	1	0.5406(4)	0.54571(16)	0.2467(6)	0.0110(5)
O6	1	0.3354(4)	0.63184(15)	0.7611(6)	0.0103(5)
O7	1	0.1888(4)	0.68277(15)	0.2021(5)	0.0096(5)
O8	1	0.0094(4)	0.65832(16)	0.7660(6)	0.0103(5)

a *U*_*eq*_ = (1/3)­{*U*_11_(aa*)^2^ + *U*_22_(bb*)^2^ + *U*_33_(cc*)^2^ + 2*U*_12_ a* b* *ab cos γ* + 2*U*_13_ a* c* *ac cos β* + 2*U*_23_ b* c* *bc cos α*}.

Figure 2 shows the crystal structure of Ba_2_LiAlSi_2_O_8_, drawn using VESTA.^39^ The Wyckoff position is only a general 4*a* site in the space group of *Pna*2_1_. The crystal structure comprises corner-sharing tetrahedra of LiO_4_, AlO_4_, and SiO_4_, which are orderly arranged in the framework, and the tetrahedra align along the *c* axis. SiO_4_ tetrahedra are isolated with other SiO_4_ tetrahedra in the structure, indicating it is a member of the nesosilicate group. Al and Si often occupied the same crystallographic sites in aluminosilicates; however, Al and Si occupied different crystallographic sites in Ba_2_LiAlSi_2_O_8_.

**Figure 2:**
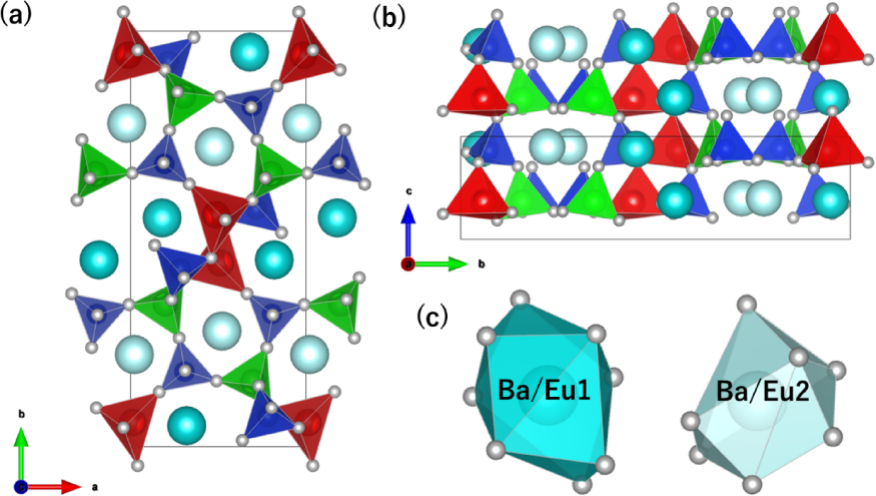
Crystal structure viewed from (a) the *c* axis and (b) the *a* axis, and polyhedra of (c) Ba/Eu1- and Ba/Eu2-site of Ba_1.96_Eu_0.04_LiAlSi_2_O_8_. Black solid line represents the unit cell. Cyan and light cyan spheres are Ba/Eu1 and Ba/Eu2 sites. Red, green, and blue tetrahedra are LiO_4_, AlO_4_, and SiO_4_. Light gray spheres represent O atoms.

To investigate in detail the distribution of Al and Si, which have similar X-ray atomic scattering factors, the cation arrangement at the tetrahedral sites was additionally examined using DFT calculations. Twelve structure models were constructed by placing Li, Al, and Si ions at four tetrahedral sites (T1, T2, T3, and T4). The lattice constants and internal coordinates were optimized via DFT calculations to minimize total energy. Table 3 shows the cation arrangements and relative DFT energies of the four lowest-energy structure models, as well as the *R*_1_ and *wR*_2_ values obtained from the XRD structural analysis. Other cation arrangements were excluded because they had higher relative DFT energies. The lowest-energy arrangement (Li, Al, Si, Si) was identical to the arrangement obtained from the XRD analysis. The arrangement with Li and Al swapped (Al, Li, Si, Si) had the second-lowest energy. However, its energy was 0.997 eV/formula-unit higher than that of the lowest-energy structure. The configuration entropy of a completely random arrangement is −4 k_B_ (0.25 log 0.25 + 0.25 log 0.25 + 0.5 log 0.5) = 0.156 meV/K formula-unit, where k_B_ denotes the Boltzmann constant. This corresponds to an energy of 0.206 eV at 1050 °C. The difference between the lowest and second-lowest energies was significantly larger than the configuration entropy. Therefore, the cations were ordered at the tetrahedral sites, and the degree of disordering should be small. An XRD analysis was also attempted for this cation arrangement, and the *R*_1_ and *wR*_2_ values were unacceptably large. The arrangement with the third-lowest energy was (Li, Si, Si, Al), and the arrangement with the fourth-lowest one was (Li, Si, Al, Si), which had swapped Al and Si compared with the arrangement with the lowest energy. Because Al and Si are difficult to distinguish in an XRD analysis, the *R*_1_ and *wR*_2_ values obtained for these two arrangements were comparable to those of the lowest-energy structure. However, DFT calculations showed higher energies of 1.203 and 1.253 eV/formula-unit for these structures, respectively. Thus, the DFT calculation results were consistent with the ordered cation arrangement obtained via XRD analysis.

**Table 3:** Cation Arrangement at Tetrahedral Sites, Relative Energies Evaluated by DFT Calculations, and XRD Analysis Results for the Four Lowest-Energy Cation Arrangements

No.	T1	T2	T3	T4	Δ*E* (eV/formula-unit)	*R*_1_ (%)	*wR*_2_ (%)
1	Li	Al	Si	Si	0	2.41	3.71
2	Al	Li	Si	Si	0.997	9.44	23.81
3	Li	Si	Si	Al	1.203	2.46	3.96
4	Li	Si	Al	Si	1.253	2.47	4.01

Considering the tetrahedral framework of Ba_2_LiAlSi_2_O_8_, each LiO_4_ tetrahedron shares corners with two LiO_4_ units, one AlO_4_ unit, and four SiO_4_ units, whereas each AlO_4_ tetrahedron shares corners with one LiO_4_ unit and four SiO_4_ units. From the electrostatic repulsion viewpoint, the LiO_4_ tetrahedron shares corners with a greater number of surrounding tetrahedra than the AlO_4_ tetrahedron due to the lower valence state of Li^+^. Ba occupies two polyhedral sites formed by the tetrahedral framework, where the Ba1 and Ba2 sites are coordinated by eight and seven oxygen atoms, respectively. Table S2 summarizes the cation–anion distances and bond valence sums (BVS).^40^ The average Ba–O distances for the Ba1 and Ba2 sites are 2.87 and 2.79 Å, respectively. The BVS values for Ba1 and Ba2 are 1.88 and 1.91, respectively. The average bond lengths of Li–O, Al–O, Si1–O, and Si2–O are 2.00, 1.75, 1.63, and 1.63 Å, respectively. These values correspond to the order of the ionic radii of Li^+^ (0.59 Å), Al^3+^ (0.39 Å), and Si^4+^ (0.26 Å). The BVS values for the Li, Al, Si1, and Si2 sites are 0.95, 3.07, 3.99, and 3.91, respectively. From the BVS values, it is clearly confirmed that Li^+^, Al^3+^, and Si^4+^ are orderly arranged at the tetrahedral sites.

The crystal structure of Ba_2_LiAlSi_2_O_8_ is related to those of PbZnSiO_4_ (larsenite) and PbLiPO_4_.^41,42^ Considering the crystal structure of Ba_2_LiAlSi_2_O_8_ based on that of PbZnSiO_4_, the Pb site is substituted by Ba and the Zn site is orderly substituted by Li and Al in a 1:1 ratio. Based on that of PbLiPO_4_, the Pb site is substituted by Ba and half of the Li site is orderly substituted by Al. The PO_4_ unit is substituted by the SiO_4_ unit. Figure 3 shows the phase diagram of the BaO-Li_2_O–Al_2_O_3_–SiO_2_ quasi-quaternary system. Li_7_Ba_3_Al_3_O_11_, LiBa_2_AlO_4_ (Li–Ba–Al–O system), Li_2_BaSiO_4_ (Li–Ba–Si–O system), Ba_13_Al_22_Si_10_O_66_, BaAl_2_Si_2_O_8_ (Ba–Al–Si–O system), LiAlSiO_4_, LiAlSi_2_O_6_, and LiAlSi_4_O_10_ (Li–Al–Si–O system) are known crystal phases of the quasi-ternary system.^43–50^ In summary, single-crystal XRD analysis and DFT calculations revealed that a new crystal phase, Ba_2_LiAlSi_2_O_8_, was identified in the BaO-Li_2_O–Al_2_O_3_–SiO_2_ quasi-quaternary system.

**Figure 3:**
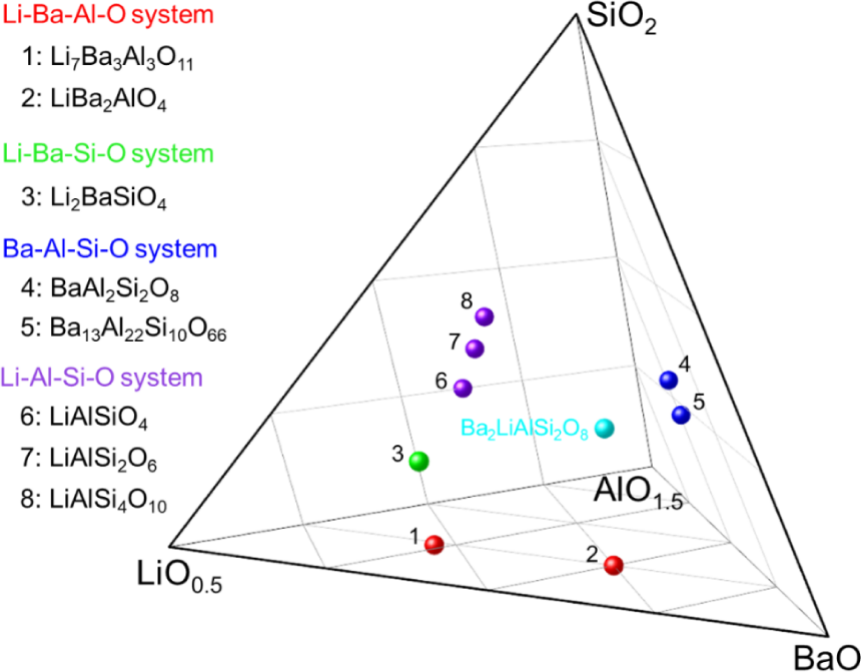
Phase diagram of the BaO–Li_2_O–Al_2_O_3_–SiO_2_ quasi-quaternary system.

### 3.2 Powder Synthesis and Luminescence Properties of Ba_2_LiAlSi_2_O_8_: Eu^2+^ Phosphors

To evaluate the luminescence properties of powder phosphors and fabricate the prototype of pc-LEDs, powder synthesis was performed based on the Ba_2_LiAlSi_2_O_8_:Eu^2+^ composition. Figure 4 shows the powder XRD patterns of Ba_2(1–*x*)_Eu_2*x*_LiAlSi_2_O_8_ (*x* = 0, 0.005, 0.02, 0.04, and 0.06). The XRD patterns of all samples are consistent with the diffraction patterns calculated from the crystal structure determined by single-crystal XRD analysis. The crystal phase of Ba_2_LiAlSi_2_O_8_ was mainly obtained in all samples. Figure 5 shows the lattice parameters of Ba_2(1–*x*)_Eu_2*x*_LiAlSi_2_O_8_ (*x* = 0, 0.005, 0.02, 0.04, and 0.06) phosphors obtained by whole pattern fitting. The linear decrease in the lattice parameters with an increase in Eu content is attributed to the difference in the ionic radii between Ba^2+^ (*r* ^VIII^ = 1.42 Å) and Eu^2+^ (*r* ^VIII^ = 1.25 Å),^51^ which produces the lattice distortion.^52^ Table 4 summarizes the cation contents obtained by ICP analysis. The experimental result (Ba:Eu:Li:Al:Si = 1.91:0.08:1.00:1.00:2.00) corresponds to the theoretical atomic ratio value of Ba_2(1–*x*)_Eu_2*x*_LiAlSi_2_O_8_ (*x* = 0.04).

**Figure 4:**
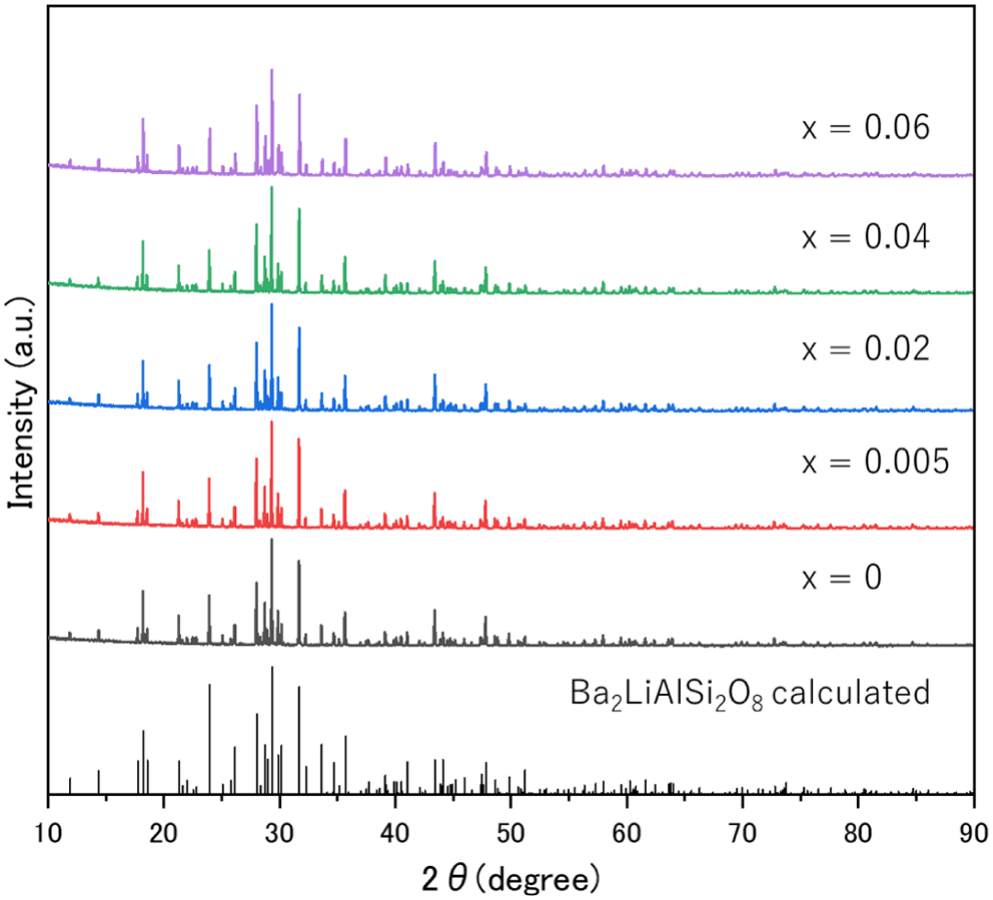
XRD patterns of Ba_2(1–*x*)_Eu_2*x*_LiAlSi_2_O_8_ (*x* = 0, 0.005, 0.02, 0.04, and 0.06) phosphors.

**Figure 5:**
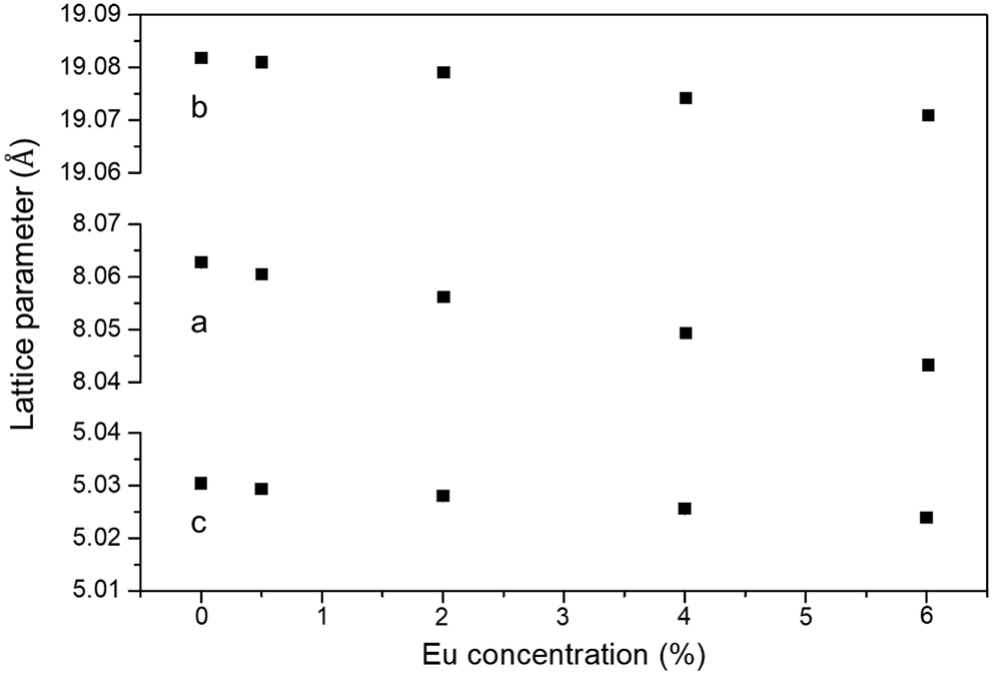
Lattice constant of Ba_2(1–*x*)_Eu_2*x*_LiAlSi_2_O_8_ (*x* = 0, 0.005, 0.02, 0.04, and 0.06) phosphors.

**Table 4:** Cation Composition of Ba_2(1–*x*)_Eu_2*x*_LiAlSi_2_O_8_ (*X* = 0.04) Phosphors by ICP Analysis^a^

	Ba	Eu	Li	Al	Si	O	Total
Theoretical (wt %)	53.4	2.46	1.41	5.46	11.4	25.87	100.0
ICP result (wt %)	52.7	2.43	1.40	5.43	11.3	25.71^b^	98.9
Mol ratio of ICP result (fixed Al to 1 mol)	1.91	0.08	1.00	1	2.00		

a The molar ratio of cation is also listed.

b This value was calculated from the metal element contents.

Figure 6 shows the excitation and emission spectra of Ba_2(1–*x*)_Eu_2*x*_LiAlSi_2_O_8_ (*x* = 0.005, 0.02, 0.04, and 0.06) phosphors. The Eu concentration optimized to show the highest intensity was given by *x* = 0.04. In the *x* = 0.04 sample, the broadband excitation spectrum is shown from 200 to 450 nm while the emission spectrum shows a peak at 497 nm with a full width at half-maximum of 85 nm under 372 nm excitation, which originated from the 5d–4f transition of Eu^2+^ in the Ba_2_LiAlSi_2_O_8_ host material. With an increase in Eu content, the excitation band intensity at wavelengths longer than 400 nm increased and the emission peak position slightly shifted to longer wavelengths. The excitation and emission intensities gradually increased with Eu content and finally decreased in the sample with *x* = 0.06 due to the concentration quenching. No significant change in the emission peak was observed for this phosphor even after prolonged UV excitation for 5 h.^53^ The IQE and EQE of the Ba_2(1–*x*)_Eu_2*x*_LiAlSi_2_O_8_ (*x* = 0.04) phosphor obtained by 372 nm excitation were 51.0% and 42.7%, respectively.

**Figure 6:**
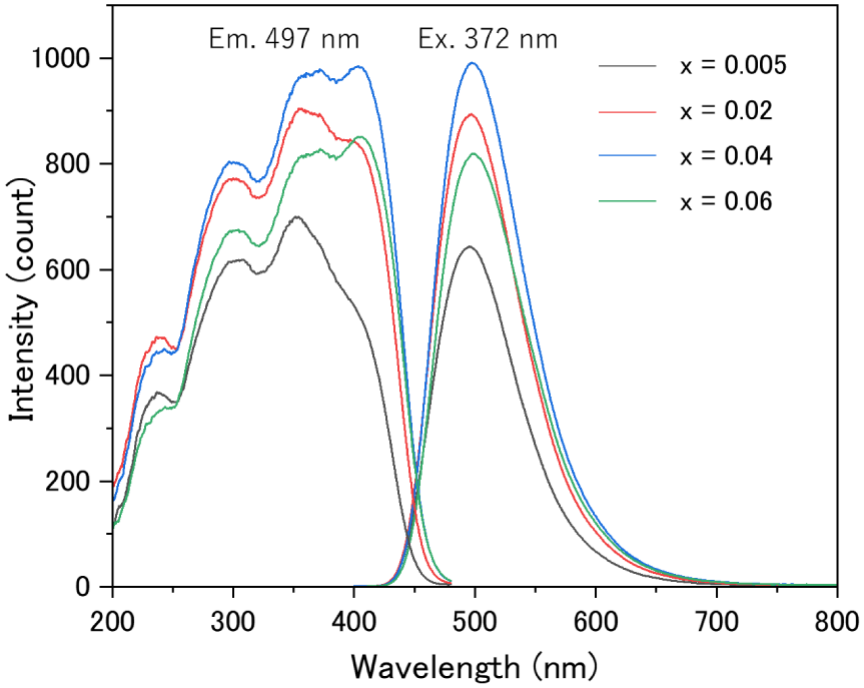
Excitation (λ_em_ = 497 nm) and emission (λ_ex_ = 372 nm) spectra of Ba_2(1–*x*)_Eu_2*x*_LiAlSi_2_O_8_ (*x* = 0.005, 0.02, 0.04, and 0.06) phosphors.

Figure 7 shows the luminescence decay curve of the Ba_2(1–*x*)_Eu_2*x*_LiAlSi_2_O_8_ (*x* = 0.04) phosphor. The lifetime was obtained by fitting the decay curve with a biexponential function corresponding to two Eu^2+^ sites in the crystal structure and a background term. The decay components were determined to be 0.19 and 0.58 μs, with relative weights of 1247 and 8691, respectively. The average decay time was calculated to be 0.56 μs, which is consistent with typical values for the 5d–4f transitions of Eu^2+^.

**Figure 7:**
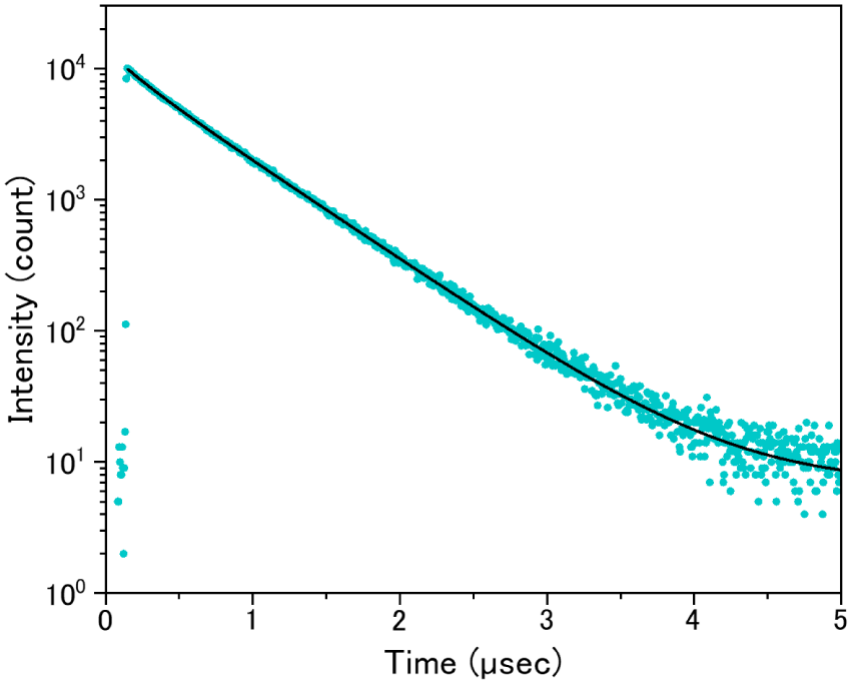
Measured (light-blue dot) and fitted (black line) decay curves of Ba_2(1–*x*)_Eu_2*x*_LiAlSi_2_O_8_ (*x* = 0.04) phosphor.

Figure 8a and b shows the temperature-dependent luminescence spectra and intensity of the Ba_2(1–*x*)_Eu_2*x*_LiAlSi_2_O_8_ (*x* = 0.04) phosphor. Figure S4 shows temperature-dependent normalized luminescence spectra. The photoluminescence intensity gradually decreased with increasing temperature from 25 to 300 °C due to the thermal quenching effect. At 150 °C, the peak and integrated intensities were 67% and 70% of those at room temperature, respectively. There is little difference between changes in peak and integrated intensities, indicating that the peak shape changes slightly. No additional peaks or noticeable peak shifts were observed. These results are favorable for LED applications with no chromatic shift.

**Figure 8:**
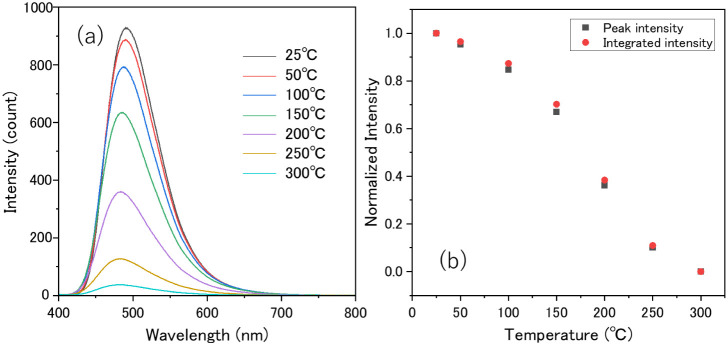
(a) Temperature-dependent luminescence spectra, (b) peak and integrated intensities of Ba_2(1–*x*)_Eu_2*x*_LiAlSi_2_O_8_ (*x* = 0.04) phosphor under 372 nm excitation.

### 3.3 Electroluminescence of Blue–Green Emitting pc-LEDs with Ba_2_LiAlSi_2_O_8_:Eu^2+^ Phosphor

To investigate the potential of the Ba_2(1–*x*)_Eu_2*x*_LiAlSi_2_O_8_ (*x* = 0.04) phosphor, a pc-LED with 405 nm LED chip was fabricated. Figure 9 shows the EL spectrum of the blue–green emitting LED with 405 nm LED chip under forward-bias currents from 10 to 60 mA. The inset shows the pc-LED emission when the current is turned on. The EL spectra show the emission band at around 400 nm derived from the LED chip and the blue–green emission band of Ba_2_LiAlSi_2_O_8_: Eu^2+^ phosphor at around 500 nm. The CIE chromaticity coordinates of the LED are shown in Figure 10. With an increase in current, the CIE chromaticity coordinates were slightly shifted in the blue–green region. The blue–green phosphor developed in this study has the potential to fill the cyan gap in white LEDs. In addition, the color coordinated for an automated driving system (ADS) marker lamp is regulated in the blue–green region of (CIE *x*, CIE *y*) = (0.012, 0.495), (0.200, 0.400), (0.200, 0.320), and (0.040, 0.320) by the SAE. Finally, we emphasized that the novel blue–green Ba_2_LiAlSi_2_O_8_: Eu^2+^ phosphor has potential for exterior ADS marker lights and future pc-LED applications.

**Figure 9:**
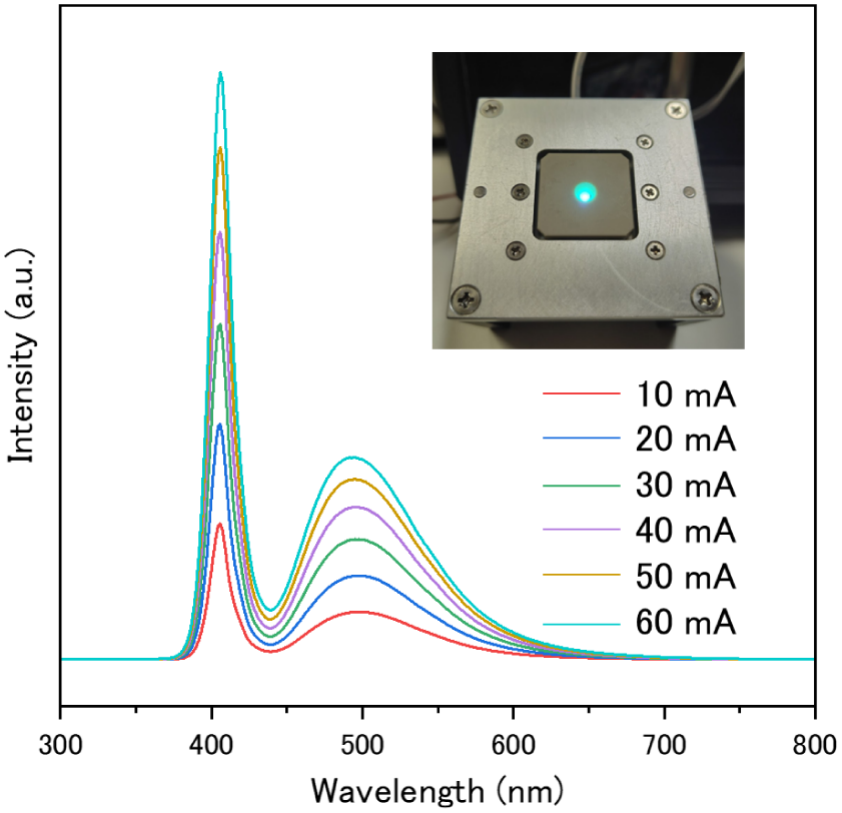
EL spectra of blue–green emitting LEDs with 405 nm LED chips and Ba_2(1–*x*)_Eu_2*x*_LiAlSi_2_O_8_ (*x* = 0.04) phosphor. The inset shows the pc-LED emission observed when the current is turned on.

**Figure 10:**
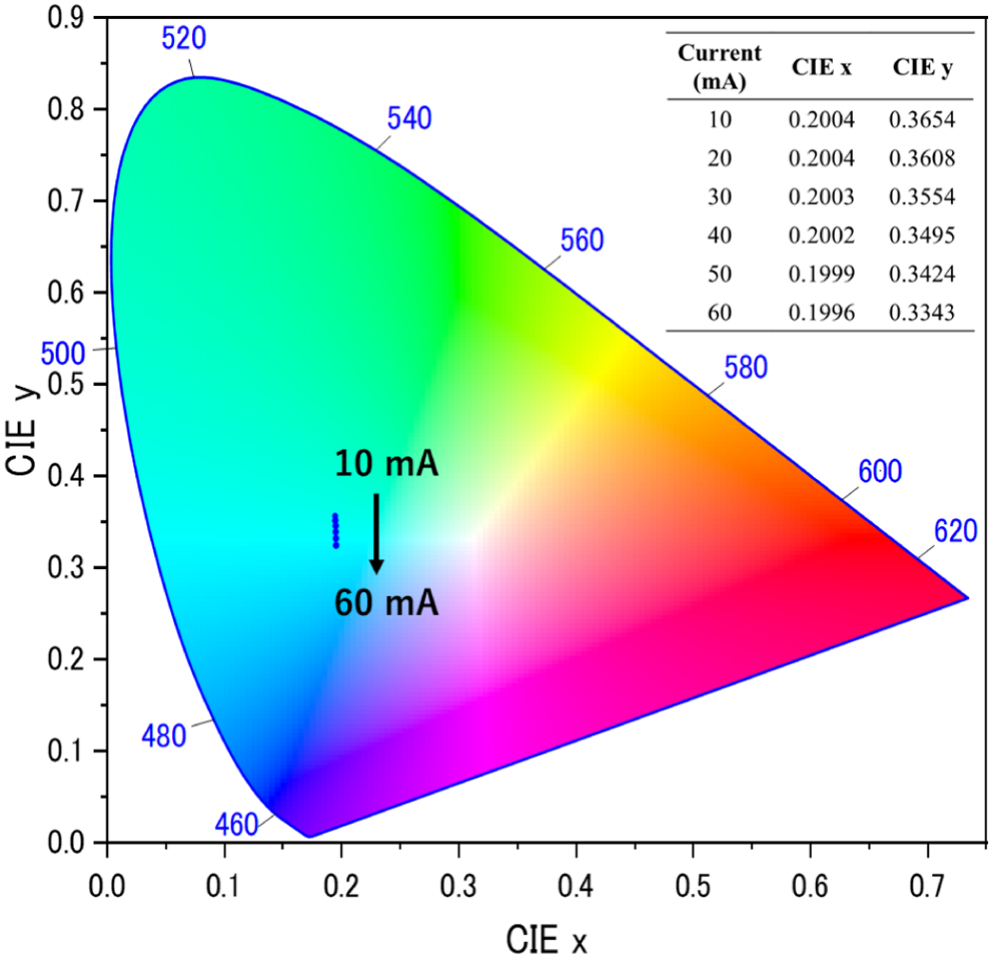
CIE chromaticity coordinates of pc-LED.

## 4 Conclusion

A new oxide-based blue–green emitting Ba_2_LiAlSi_2_O_8_:Eu^2+^ phosphor was discovered using a single-particle-diagnosis approach. The crystal structure, which is related to those of PbZnSiO_4_ (larsenite) and PbLiPO_4_, was revealed via single-crystal XRD analysis. DFT calculations corroborated the Al and Si arrangement obtained from the single-crystal XRD analysis results. Powder phosphor of Ba_2_LiAlSi_2_O_8_:Eu^2+^ was successfully obtained via a solid-state reaction. Ba_2(1–*x*)_Eu_2*x*_LiAlSi_2_O_8_ (*x* = 0.04) phosphor showed a blue–green emission under 372 nm excitation. The IQE and EQE were 51.0% and 42.7%, respectively. The peak intensity at 150 °C was 67% of that at room temperature. The EL spectra of the corresponding pc-LEDs showed a blue–green emission band, and the CIE chromaticity coordinates were in the blue–green region regulated by the SAE, indicating that the blue–green emitting Ba_2_LiAlSi_2_O_8_:Eu^2+^ phosphor has potential applications in exterior ADS marker lights and future LED technologies. The single-particle-diagnosis approach is effective for discovering new phosphors; thus, it will facilitate the development of pc-LEDs.

## Supplementary Material





## Notes

CIF file (Ba_1.96_Eu_0.04_LiAlSi_2_O_8_) (CIF)
Figure S1: XRD pattern of the powder product of Ba/Eu/Li/Al/Si = 39.2:0.8:10:10:40 calcined at 1050°C for 5 h in a reducing atmosphere (H_2_:N_2_ = 5:95 gas); Figure S2: excitation and emission spectra of the blue–green emitting single particle; Figure S3: EDS spectrum of the blue–green emitting single particle; Figure S4: temperature-dependent normalized luminescence spectra of Ba_2(1–x)_Eu_2x_LiAlSi_2_O_8_ (x = 0.04) phosphor under 372 nm excitation; Table S1: anisotropic displacement parameters (Å^2^) of Ba_1.96_Eu_0.04_LiAlSi_2_O_8_; Table S2: bond length, average bond length (Å) and BVS for Ba_1.96_Eu_0.04_LiAlSiO_8_ (PDF)
