# Supplementary material for: Blue–Green Emitting Phosphor Ba2LiAlSi2O8:Eu2+ for Phosphor-Converted Light-Emitting Diodes via Single-Particle Diagnosis in a Quasi-Quaternary System
Source: ACS Appl Mater Interfaces. 2026 May 14;18(20):28857–65. doi: 10.1021/acsami.6c02416 (PMC13220227; doi:10.1021/acsami.6c02416)
Supplement: Supplementary file 2 [file am6c02416_si_002.pdf]

Blue–green emitting phosphor $Ba_2LiAlSi_2O_8$: Eu^{2+} for phosphor-converted light-emitting diodes via single-particle-diagnosis in a quasi-quaternary system

*Akihiro Nakanishi[a], Shiro Funahashi[a], Yukinori Koyama[b], Hisanori Yamane[a], Kohsei Takahashi[a], Takayuki Nakanishi[a], Naoto Hirosaki[a], Takashi Takeda[a, *]*

[a] *Advanced Phosphor Group, National Institute for Materials Science, Tsukuba, Ibaraki 305-0044, Japan*

[b] *Center for Basic Research on Materials, National Institute for Materials Science, Tsukuba, Ibaraki 305-0047, Japan*

*Corresponding author, (TAKEDA.Takashi@nims.go.jp)

Supporting Information

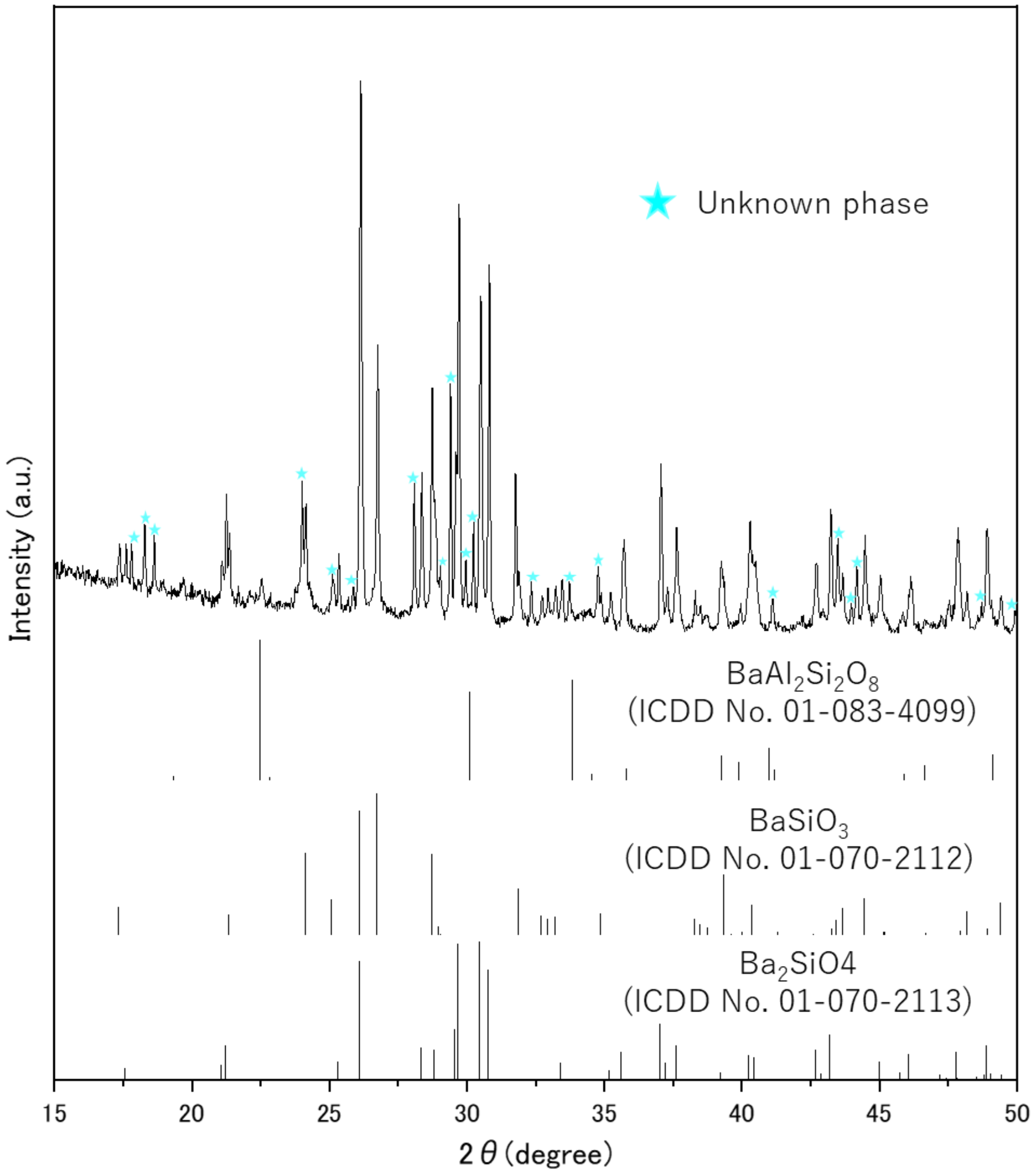

Figure S1 XRD pattern of the powder product of Ba/Eu/Li/Al/Si = 39.2:0.8:10:10:40 calcined at 1050°C for 5 h in a reducing atmosphere (H_2:N_2 = 5:95 gas).

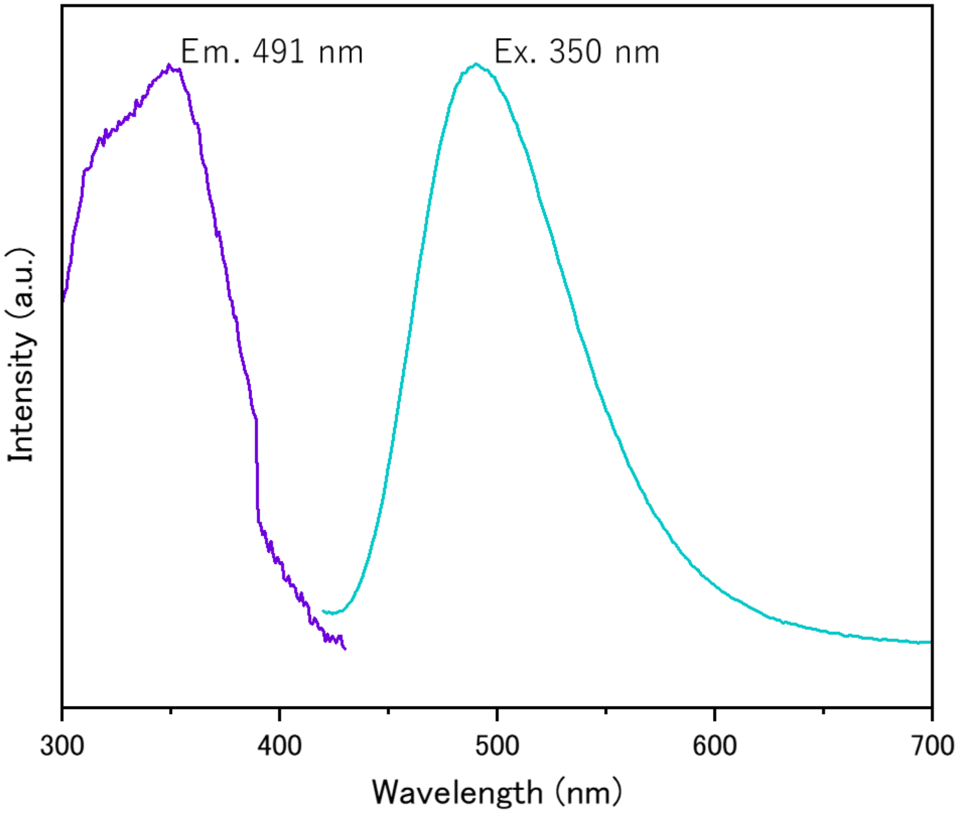

Figure S2 Excitation and emission spectra of the blue–green emitting single particle.

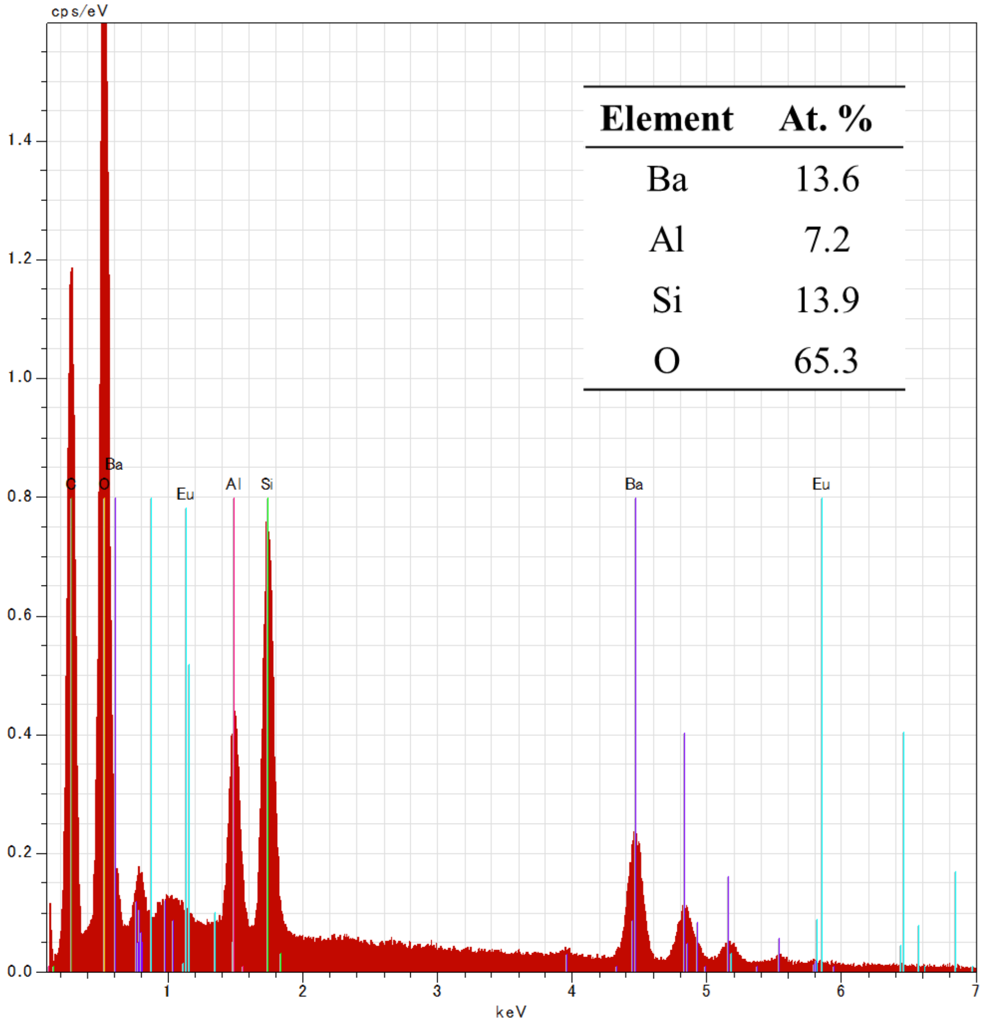

Figure S3 EDS spectrum of the blue–green emitting single particle. The cation composition is shown in the inset table.

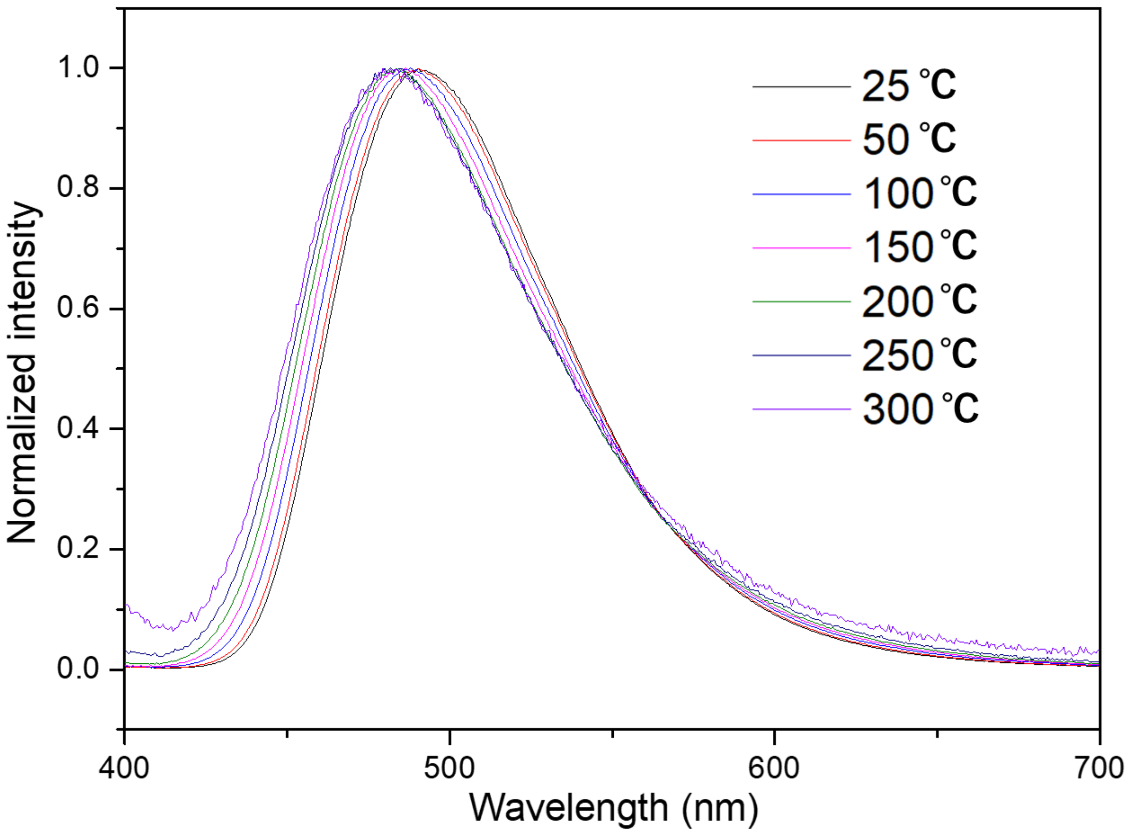

Figure S4 Temperature-dependent normalized luminescence spectra of $Ba_{2(1-x)}Eu_{2x}LiAlSi_2O_8$ (x = 0.04) phosphor under 372 nm excitation.

Table S1 Anisotropic displacement parameters ($Å^2$) of $Ba_{1.96}Eu_{0.04}LiAlSi_2O_8$

Atom	U_{11}	U_{22}	U_{33}	U_{12}	U_{13}	U_{23}
Ba/Eu1	0.01100 (8)	0.00765 (7)	0.00893 (7)	−0.00135 (6)	0.00008 (10)	0.00042 (10)
Ba/Eu2	0.00618 (7)	0.01255 (7)	0.00875 (7)	−0.00199 (6)	0.00037 (11)	−0.00037 (10)
Li	0.008 (2)	0.014 (3)	0.014 (3)	0.001 (2)	−0.001 (3)	0.000 (3)
Al	0.0049 (4)	0.0051 (3)	0.0063 (5)	−0.0008 (3)	0.0004 (4)	−0.0004 (4)
Si1	0.0047 (3)	0.0053 (3)	0.0059 (3)	0.0001 (2)	−0.0007 (5)	−0.0008 (5)
Si2	0.0063 (4)	0.0053 (3)	0.0047 (4)	−0.0007 (3)	−0.0007 (4)	−0.0004 (4)
O1	0.0116 (11)	0.0105 (10)	0.0147 (15)	0.0044 (9)	−0.0006 (10)	−0.0017 (9)
O2	0.0082 (12)	0.0088 (11)	0.0056 (10)	−0.0033 (9)	0.0011 (9)	−0.0016 (8)
O3	0.0145 (14)	0.0092 (11)	0.0057 (10)	−0.0036 (10)	0.0007 (10)	0.0011 (8)
O4	0.0146 (14)	0.0045 (10)	0.0092 (11)	−0.0004 (9)	−0.0009 (10)	0.0005 (8)
O5	0.0095 (13)	0.0139 (12)	0.0097 (11)	−0.0046 (10)	−0.0038 (9)	0.0018 (9)
O6	0.0087 (13)	0.0089 (11)	0.0132 (12)	0.0026 (10)	0.0015 (10)	0.0003 (9)
O7	0.0114 (13)	0.0105 (11)	0.0071 (10)	−0.0002 (10)	−0.0001 (9)	0.0006 (8)
O8	0.0048 (12)	0.0134 (12)	0.0127 (12)	−0.0020 (10)	−0.0011 (9)	0.0011 (9)

Table S2 Bond length, average bond length (Å) and BVS for $Ba_{1.96}Eu_{0.04}LiAlSiO_8$.

Atom		Bond length	Average bond length	BVS
Ba/Eu1	-O1^{i}	2.654(3)	2.87	1.88
	-O1^{ii}	2.684(3)		
	-O1^{iii}	2.967(3)		
	-O2^{iii}	3.077(3)		
	-O5	2.976(3)		
	-O6	2.769(3)		
	-O7	2.671(3)		
	-O8	3.146(3)		

	-O3	2.903(3)		
	-O4^{iv}	2.727(3)		
	-O4	2.880(3)		
Ba/Eu2	-O6	2.746(3)	2.79	1.91
	-O7^{iv}	2.678(3)		
	-O7	2.637(3)		
	-O8	2.943(3)		
	-O2	2.020(8)		
Li	-O5^{ii}	2.077(8)	2.00	0.95
	-O5^{v}	1.980(10)		
	-O6	1.931(8)		
	-O2	1.749(3)		
Al	-O3^{v}	1.753(3)	1.75	3.07
	-O4^{iv}	1.747(3)		
	-O8^{vi}	1.745(3)		
	-O4	1.656(3)		
Si1	-O6	1.600(3)	1.63	3.99
	-O7^{v}	1.606(3)		
	-O8	1.640(3)		
	-O1	1.597(3)		
Si2	-O2	1.659(3)	1.63	3.91
	-O3	1.667(3)		
	-O5	1.613(3)		

Symmetry code: (i) $x-1, y, z$; (ii) $-x+1, -y+1, z+1/2$; (iii) $-x+1, -y+1, z-1/2$; (iv) $x+1/2, -y+3/2, z$; (v) $x, y, z+1$; (vi) $x+1, y, z$.
